# Synthesis and Biological Evaluation of Substituted Desloratadines as Potent Arginine Vasopressin V2 Receptor Antagonists

**DOI:** 10.3390/molecules19022694

**Published:** 2014-02-24

**Authors:** Shuai Mu, Ying Liu, Min Gong, Deng-Ke Liu, Chang-Xiao Liu

**Affiliations:** 1 School of Chemical Engineering and Technology, Tianjin University, Tianjin 300072, China; E-Mail: mushuai2005@163.com; 2 Tianjin Key Laboratory of Molecular Design and Drug Discovery, Tianjin Institute of Pharmaceutical Research, Tianjin 300193, China; E-Mails: liuy6@tjipr.com (Y.L.); gongm@tjipr.com (M.G.); liudk@tianjinipr.com (D.-K.L.); 3 State Key Laboratory of Drug Delivery Technology and Pharmacokinetics, Tianjin Institute of Pharmaceutical Research, Tianjin 300193, China

**Keywords:** substituted desloratadine, synthesis, arginine vasopressin receptor antagonists, biological activity

## Abstract

Twenty-one non-peptide substituted desloratadine class compounds were synthesized as novel arginine vasopressin receptor antagonists from desloratadine via successive acylation, reduction and acylation reactions. Their structures were characterized by ^1^H-NMR and HRMS, their biological activity was evaluated by *in vitro* and *in vivo* studies. The *in vitro* binding assay and cAMP accumulation assay indicated that these compounds are potent selective V2 receptor antagonists. Among them compounds **1n**, **1t** and **1v** exhibited both high affinity and promising selectivity for V2 receptors. The *in vivo* diuretic assay demonstrated that **1t** presented remarkable diuretic activity. In conclusion, **1t** is a potent novel AVP V2 receptor antagonist candidate.

## 1. Introduction

Arginine vasopressin (AVP), a neurohypophysial peptide hormone that is secreted mainly from the posterior pituitary gland in response to low blood volume or high serum osmolality exerts its biological action through three major G-protein-coupled receptors, V1a, V1b and V2 [1,2,3]. The V2 receptors, which are localized predominately in the kidney collecting tubules, are responsible for controlling water reabsorption and salt (NaCl) balance [4]. The receptor stimulates adenylate cyclase, which results in the production of cyclic AMP [5]. Thus, there is potential to develop a vasopressin V2 receptor antagonist for the treatment of disorders such as congestive heart failure [6,7,8,9], hypertension [10,11], renal disease [12,13], edema [14,15], liver cirrhosis [16,17], hyponatremia [18,19,20,21,22], inappropriate antidiuretic hormone secretion (SIADH) syndrome [23] and any state involving excessive retention of water.

**Figure 1:**
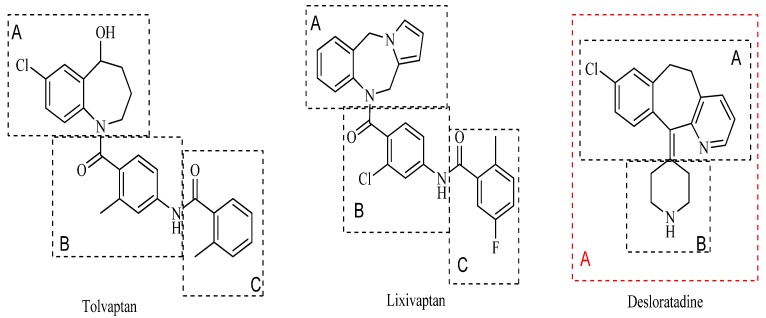
Chemical structures of tolvaptan, lixivaptan and desloratadine (**black**: previous study; **red**: reported here in this article).

Numerous AVP receptor antagonists were developed and evaluated in recent decades [24,25,26,27,28,29,30]. A few of them have undergone sufficient clinical trials to be on the market, such as the dual V1a/V2 receptor antagonist conivaptan and the selective V2 receptor antagonist tolvaptan approved for the treatment of hyponatremia in the USA. Another promising selective AVP V2 receptor antagonist, lixivaptan, is still undergoing phase 3 clinical trials at this moment. The structures of most extant AVP receptor antagonists include a benzene-fused seven membered ring system (ring A) and two aromatic rings (ring B and ring C) linked through amide bonds. Recently, we reported some amide and sulfamide derivatives of desloratadine, which are potent AVP V2 receptor antagonists [31]. Desloratadine is a selective, H_1_-receptor antagonist, and also has anti-inflammatory activity [32]. In the previous study, ring A and ring B of classic V2 receptor antagonists were replaced by desloratadine (Figure 1). In a continuous study, we synthesized several compounds centered on a desloratadine scaffold as ring A (compounds **1a**, **1b**, **1h** and **1i**, see Table 1) and found that they exhibited potent diuretic activity [33]. Therefore, additional compounds with a desloratadine scaffold as ring A were designed, synthesized and evaluated. Herein, we report the synthesis and biological evaluation of this series of substituted desloratadine designed as potent AVP V2 receptor agonists.

**Table 1:** The structures of the target compounds and their biological activity evaluation. 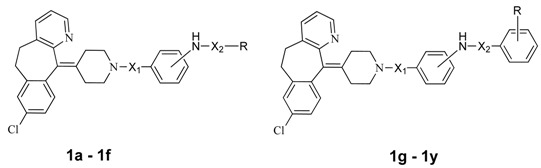

Compound	Structures of Target Compound				Bingding Assy (IC50; nmol/mL)		cAMP Assay (V2, IC50; nmol/mL)	Volume of Urine (mL, 0–20 h)
	X_1_	X_2_	Position Of -NH_2_	R	V2	V1a		
**1a**	CO	CO	*p*-NH_2_	-CH_2_CH_3_	6.3	130	26	27.5±5.7
**1b**	CO	CO	*p*-NH_2_	-CH_2_CH_2_CH_3_	47	>1000		
**1c**	CO	CO	*p*-NH_2_	-CH_2_CH_2_CH_2_Cl	25	>1000		
**1d**	CO	SO_2_	*p*-NH_2_	-CH_2_CH_3_	11	92	160	21.4±4.1
**1e**	SO_2_	CO	m-NH_2_	-CH_2_CH_2_CH_3_	26	176		
**1f**	SO_2_	CO	*p*-NH_2_	-CH_2_CH_2_CH_3_	40	480		
**1g**	CO	CO	*p*-NH_2_	H	19	330		
**1h**	CO	CO	*p*-NH_2_	2-Me	23	210		
**1i**	CO	CO	*p*-NH_2_	4-Me	18	220		
**1j**	CO	CO	*p*-NH_2_	3-Me	15	370	53	19.3 ± 5.5
**1k**	CO	CO	*p*-NH_2_	2-Cl	27	490		
**1l**	CO	CO	*p*-NH_2_	3-Cl	16	560		
**1m**	CO	CO	*p*-NH_2_	2-F	20	170		
**1n**	CO	CO	*p*-NH_2_	3-OMe	8.5	390	380	19.9 ± 6.7
**1o**	CO	CO	*p*-NH_2_	3-NO_2_	18	550		
**1p**	CO	CO	*p*-NH_2_	4-NO_2_	52	>1000		
**1q**	CO	SO_2_	*p*-NH_2_	4-Me	24	720		
**1r**	CO	SO_2_	*p*-NH_2_	2-Cl	11	830	220	16.1 ± 3.2
**1s**	CO	SO_2_	*p*-NH_2_	2,5-DiCl	9.2	320	37	18.3 ± 4.3
**1t**	SO_2_	CO	*m*-NH_2_	H	7.7	>1000	98	28.1 ± 5.0
**1u**	SO_2_	CO	*m*-NH_2_	3-Cl	19	840		
**1v**	SO_2_	CO	*m*-NH_2_	3- NO_2_	5.5	630	110	11.9 ± 2.7
**1w**	SO_2_	SO_2_	*m*-NH_2_	4-Me	30	860		
**1x**	SO_2_	CO	*p*-NH_2_	3-Cl	310	>1000		
**1y**	SO_2_	SO_2_	*p*-NH_2_	4-Me	170	920		
Control								6.5 ± 0.5
tolvaptan								28.0 ± 6.5

## 2. Results and Discussion

**Scheme 1:**
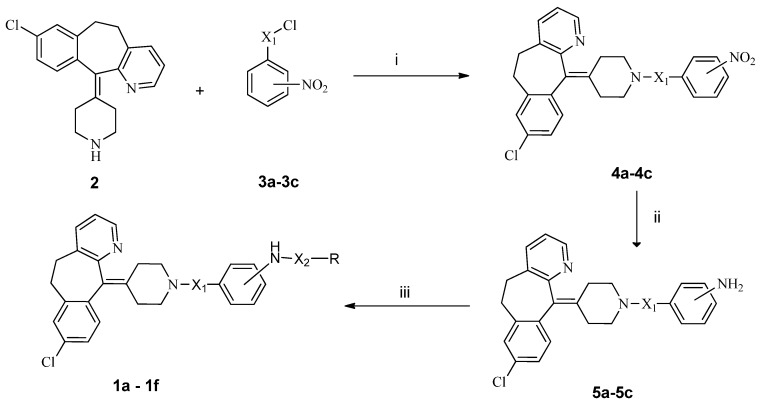
Synthetic route to **1a**–**f**.

**Scheme 2:**
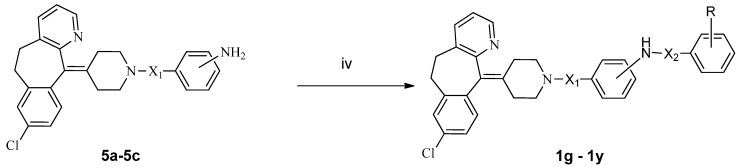
Synthetic route to **1g**–**y**.

The synthetic routes used in this study are illustrated in Scheme 1 and Scheme 2, respectively. As shown in Scheme 1, the acylation of **2** with a *p*-nitrobenzoyl chloride, *p*-nitrobenzene sulfonyl chloride or *m*-nitrobenzene sulfonyl chloride provided **4a**–**c**, which were subsequently reduced with SnCl_2_ to provide the corresponding anilines **5a**–**c** in satisfied yields. Acylation of **5a**–**c** with alkyl chloride or alkylsulfonyl chloride yielded the target compounds **1a**–**f**. Similarly, as shown in Scheme 2, acylation of **5a**–**c** with substituted benzoyl chlorides or benzenesulfonyl chlorides gave target product **1g**–**y**.

*Reagents and Conditions*: i: DCM, TEA, 0 °C. ii: SnCl_2 _and concentrated hydrochloric acid in refluxing ethanol; iii: Alkyl chloride or alkylsulfonyl chloride; DCM, TEA, 0 °C.

*Reagents and Conditions*: iv. Substituted benzoyl chloride or benzenesulfonyl chloride, DCM, TEA, 0 °C.

Twenty-one compounds **1c**–**g**, **1j**–**y** were synthesized and characterized by ^1^H-NMR and HRMS. In order to provide a comprehensive understanding of the structure-activity relationships, compounds **1a**, **1b**, **1h** and **1i** were included in this research as well. The structures of the target compounds **1a**–**y** and evaluation of the biological features were summarized in Table 1. The binding affinity was determined by a radioligand binding assay and cAMP assay on V1a and V2 over-expressing cells. These compounds had specific affinity to human AVP receptors. Furthermore, they showed high selectivity to V2 receptors. When ring C was replaced by an alkyl group with straight chain, their binding constants to V2 receptor were reduced significantly along the length increase of the carbon chain. Halogen-substituted alkyl group slightly increased their binding affinity to V2 receptors. When ring C was a substituted benzene ring, the different substituted positions of methyl or halogen did not significantly affect the binding affinity to V2 receptors or V1a/V2 selectivity of the compounds.

Moreover, *meta*-substituted compounds had a relatively higher binding affinity to V2 receptors than *ortho*-substituted or *para*-substituted compounds. Compounds **1x** and **1y** showed poor binding activity to both V1a and V2 receptors. Compounds **1a**, **1n**, **1t** and **1v** presented encouraging binding affinity, with both remarkable binding affinity and selectivity for the V2 receptor.

Several compounds with satisfactory binding affinity were selected to conduct the *in vivo* diuretic assay, with **1a** as the reference compound [33]. As shown in Table 1, it is evident that the selected compounds have significant diuretic activity, as they strongly increased urine volume compared with the control group. Compound **1t** exhibited an excellent diuretic activity which was equivalent to tolvaptan. It is very difficult to declare the relationship between diuretic activity and binding affinity. Because of specific differences in the vasopressin receptors, it may be difficult to draw a direct comparison between the diuretic assay in rats and the binding assay in cells expressing the human receptor.

## 3. Experimental

### 3.1. General Information

Desloratadine was purchased from Beijing Datian Fengtuo Chemistry Co., Ltd (Beijing, China). Other reagents and solvents were obtained from commercial suppliers. The human recombinant vasopressin V1a (Cat. ES-361-C) and V2 (ES-363-C) receptors in 1321N1 host cell were obtained from Perkin Elmer Inc (Waltham, MA, USA). The Sprague-Dawley rats were purchased from Tianjin Shanchuanhong Experimental Animals Co., Ltd (Tianjin, China). All reactions were monitored by thin layer chromatography. Silica gel chromatography was conducted on a Teledyne Isco COMBIFLASH Rf200 Purification System (Teledyne Isco, Inc., Lincoln, NE, USA) (petroleum ether and ethyl acetate, gradient elution). HPLC data was obtained with an Agilent 1260 (Agilent Technologies, Inc., Santa Clara, CA, USA) equipped with a Grace C18 column (5 μm, 250 mm × 4.6 mm, Lot No. 55/182). ^1^H-NMR spectra were recorded on a Bruker AV400 NMR (Bruker, Billerica, MA, USA) and HRMS were measured on a VG ZAB-HS instrument (VG Instruments, London, UK). Melting points (uncorrected) were determined on a YRT-3 Melting Point Tester (Precision Instrument of Tianjin University, Tianjin, China).

### 3.2. Synthesis

*(4-(8-Chloro-5H-benzo[5,6]cyclohepta[1,2-b]pyridin-11(6H)-ylidene)piperidin-1-yl)(4-nitrophenyl) methanone* (**4a**) [33]. To a solution of desloratadine (100 g, 322 mmol) in CH_2_Cl_2_ (500 mL), Et_3_N (48 g, 480 mmol) was added and the mixture was stirred at 0 °C for 10 min. Then **2a** (59.8 g, 322 mmol) dissolved in CH_2_Cl_2_(200 mL) was added dropwise into the mixture and stirring was continued for another 2 h. The reaction misxture was washed successively with 1 mol/L hydrochloric acid and water. The organic layer was dried over anhydrous magnesium sulfate and evaporated to give the crude product as a yellow powder, which was recrystallized from ethanol affording **4a** as a white powder. Yield: 95%; m.p.: 188.2–189.0 °C; ^1^H-NMR (400 MHz, DMSO-*d_6_*): *δ* 2.18–2.32 (m, 2H), 2.41 (br s, 1H), 2.79–2.84 (m, 2H), 3.17–3.36 (m, 6H), 3.97 (br s, 1H), 7.05–7.32 (m, 4H), 7.54–7.57 (m, 1H), 7.68 (d, *J* = 8.4 Hz, 2H), 8.25–8.36 (m, 3H).

*8-Chloro-11-(1-((3-nitrophenyl)sulfonyl)piperidin-4-ylidene)-6,11-dihydro-5H-benzo[5,6]cyclohepta-[1,2-b]pyridine* (**4b**). Compound **4b** was prepared using a similar method as for **4a**. Yield: 97%, m.p.: 221.7–222.8 °C; ^1^H-NMR (CDCl_3_): *δ* 2.40–2.41 (m, 2H), 2.50–2.57 (m, 1H), 2.62–2.68 (m, 1H), 2.71–2.86 (m, 2H), 3.09–3.15 (m, 2H), 3.22–3.36 (m, 4H), 7.01 (d, *J* = 8.0 Hz, 2H), 7.11–7.15 (m, 3H), 7.45 (d, *J* = 7.6 Hz, 1H), 7.76 (t, *J* = 8.0 Hz, 1H), 8.07–8.10 (m, 1H), 8.37 (d, *J* = 6.4 Hz, 1H), 8.44–8.59 (m, 1H), 8.59 (d, *J* = 2.0 Hz, 1H).

*8-Chloro-11-(1-((4-nitrophenyl)sulfonyl)piperidin-4-ylidene)-6,11-dihydro-5H-benzo[5,6]cyclohepta-[1,2-b]pyridine* (**4c**). Compound **4c** was prepared using a similar method as for **4a**. Yield: 88%, m.p.: >230 °C; ^1^H-NMR (CDCl_3_): *δ* 2.34–2.39 (m, 2H), 2.46–2.49 (m, 1H), 2.60–2.62 (m, 1H), 2.72–2.81 (m, 2H), 2.99–3.03 (m, 2H), 3.20–3.33 (m, 4H), 6.98 (d, *J* = 8.4 Hz, 1H), 7.05–7.13 (m, 3H), 7.39 (d, *J* = 7.6 Hz, 1H), 7.91–7.94 (m, 2H), 8.33–8.37 (m, 3H).

*(4-Aminophenyl)(4-(8-chloro-5H-benzo[5,6]cyclohepta[1,2-b]pyridin-11(6H)-ylidene)piperidin-1-yl)-methanone* (**5a**) [33]. To a mixture of **4a** (100 g, 217 mmol) dissolved in ethanol (600 mL) and concentrated hydrochloric acid (150 mL), SnCl_2_ (171 g, 760 mmol) dissolved in ethanol (400 mL) was added. After the addition was completed, the mixture was heated to reflux and stirred for 4–6 h. Then the mixture was poured into ice water and extracted with dichloromethane. The organic layer was collected and concentrated under reduced pressure to give the crude product as a brown powder. Compound **5a** was obtained by recrystallization from ethyl acetate. Yield: 82%, ^1^H-NMR (CDCl_3_): *δ* 2.38–2.56 (m, 4H), 2.75–2.87 (m, 2H), 3.20–3.42 (m, 4H), 3.82 (s, 2H), 3.94 (br s, 2H), 6.59–6.62 (m, 2H), 7.06–7.15 (m, 4H), 7.22–7.26 (m, 2H), 7.40–7.43 (dd, *J*_1_ = 1.2 Hz, *J*_2 _= 7.6 Hz, 1H), 8.37 (d, *J* = 3.6 Hz, 1H).

*3-((4-(8-Chloro-5H-benzo[5,6]cyclohepta[1,2-b]pyridin-11(6H)-ylidene)piperidin-1-yl)sulfonyl) aniline* (**5b**). Compound **5b** was prepared using a similar method as used for **5a**. Yield: 78%, m.p.: 227.9–228.8 °C; ^1^H-NMR (CDCl_3_): *δ* 2.34–2.37 (m, 2H), 2.44–2.50 (m, 1H), 2.56–2.63 (m, 1H), 2.68–2.81 (m, 2H), 2.88–2.94 (m, 2H), 3.20–3.39 (m, 4H), 3.89 (s, 1H), 6.83 (dd, *J*_1_ = 2.0 Hz, *J*_2_ = 8.0 Hz, 1H), 6.99–7.01 (m, 2H), 7.04–7.12 (m, 4H), 7.23–7.27 (m, 1H), 7.38 (dd, *J*_1_ = 1.2 Hz, *J*_2_ = 7.6 Hz, 1H), 8.34 (dd, *J*_1_ = 2.0 Hz, *J*_2_ = 4.8 Hz, 1H).

*4-((4-(8-Chloro-5H-benzo[5,6]cyclohepta[1,2-b]pyridin-11(6H)-ylidene)piperidin-1-yl)sulfonyl) aniline* (**5c**). Compound **5c** was prepared using a similar method as used for **5a**. Yield: 88%, m.p.: 110.1–111.6 °C; ^1^H-NMR (CDCl_3_): *δ* 2.30–2.36 (m, 2H), 2.43–2.46 (m, 1H), 2.56–2.60 (m, 1H), 2.70–2.90 (m, 4H), 3.17–3.30 (m, 4H), 6.65–6.67 (m, 2H), 6.99–7.11 (m, 4H), 7.38 (dd, *J*_1_ = 1.6 Hz, *J*_2_ = 8.0 Hz, 1H), 7.50 (dd, *J*_1_ = 1.8 Hz, *J*_2_ = 6.6 Hz, 2H), 8.35 (dd, *J*_1_ = 1.6 Hz, *J*_2_ = 4.8 Hz, 1H).

*4-Chloro-N-(4-(4-(8-chloro-5H-benzo[5,6]cyclohepta[1,2-b]pyridin-11(6H)-ylidene)piperidine-1-carbonyl)phenyl)butanamide* (**1c**). The syntheses of **1a**, **1b**, **1h** and **1i** have been reported in our previous study [33]. Other target compounds were prepared using similar methods. Taking **1c** as an example, to a solution of **5a** (5.0 g, 12 mmol) in CH_2_Cl_2_ (40 mL), Et_3_N (1.8 g, 18 mmol)was added and the resulting mixture was then stirred at 0 °C for 10 min. Then 4-chlorobutanoyl chloride (1.7 g, 12 mmol) dissolved in CH_2_Cl_2_ (20 mL) was added dropwise into the mixture that was stirred for another 3 h. The reaction solution was washed successively with 1 mol/L hydrochloric acid and water. The organic layer was dried over anhydrous magnesium sulfate and concentrated to give the crude product as a yellow solid that was purified by silica gel chromatography to give compound **1c**. Yield: 85%, m.p.: 204.8–207.6 °C; ^1^H-NMR (CDCl_3_): *δ *2.15–2.20 (m, 2H), 2.29–2.64 (m, 6H), 2.77–2.90 (m, 2H), 3.25–3.44 (m, 4H), 3.61–3.69 (m, 3H), 4.15 (br s, 1H), 7.10–7.17 (m, 4H), 7.33 (d, *J* = 8.4 Hz, 2H), 7.43–7.49 (m, 3H), 7.95 (s, 1H), 8.39 (d, *J* = 2.8 Hz, 1H); HRMS (ESI): calcd for C_30_H_29_Cl_2_N_3_O_2_ [M + H]^+^ *m/z*: 534.1710, found: 534.1705.

*N-(4-(4-(8-Chloro-5H-benzo[5,6]cyclohepta[1,2-b]pyridin-11(6H)-ylidene)piperidine-1-carbonyl) phenyl)ethanesulfonamide* (**1d**). Yield: 44%, m.p.: 210.0–211.1 °C; ^1^H-NMR (CDCl_3_): *δ* 2.32–2.46 (m, 4H), 2.75–2.89 (m, 2H), 3.21–3.40 (m, 4H), 3.57 (br s, 1H), 4.13 (br s, 1H), 6.11–6.14 (m, 2H), 6.24–6.28 (d, *J* = 16.8 Hz, 2H), 6.98–7.16 (m, 5H), 7.24–7.28 (m, 2H), 7.41–7.45 (m, 3H), 8.37 (s, 1H); HRMS (ESI): calcd for C_28_H_28_ClN_3_O_3_S [M + H]^+^ *m/z*: 522.1613, found: 522.1609.

*N-(3-((4-(8-Chloro-5H-benzo[5,6]cyclohepta[1,2-b]pyridin-11(6H)-ylidene)piperidin-1-yl)sulfonyl) phenyl)butyramide* (**1e**). Yield: 50%, m.p.: 174.1–174.9 °C; ^1^H-NMR (CDCl_3_): *δ* 1.00 (t, *J* = 7.4 Hz, 3H), 1.75 (d, *J* = 7.6 Hz, 2H), 2.32–2.37 (m, 4H), 2.44–2.50 (m, 1H), 2.57–2.62 (m, 1H), 2.70–2.81 (m, 2H), 2.90 (t, *J* = 3.8 Hz, 2H), 3.22–3.31 (m, 4H), 7.00 (d, *J* = 8.0 Hz, 1H), 7.08–7.12 (m, 3H), 7.40–7.47 (m, 3h), 7.78 (s, 1H), 7.92 (d, *J* = 4.0 Hz, 1H), 8.35 (dd, *J*_1_ = 1.2 Hz, *J*_2_ = 4.8 Hz, 1H); HRMS (ESI): calcd for C_29_H_30_ClN_3_O_3_S [M + H]^+^ *m/z*: 536.1769, found: 536.1770.

*N-(4-((4-(8-Chloro-5H-benzo[5,6]cyclohepta[1,2-b]pyridin-11(6H)-ylidene)piperidin-1-yl)sulfonyl) phenyl)butyramide* (**1f**). Yield: 60%, mp: 210.0–211.1 °C; ^1^H-NMR (CDCl_3_): *δ* 1.01 (t, *J* = 7.4 Hz, 3H), 1.77 (q, *J* = 7.5 Hz, 1H), 2.31–2.39 (m, 4H), 2.44–2.47 (m, 1H), 2.57–2.59 (m, 1H), 2.70–2.89 (m, 4H), 3.23–3.30 (m, 4H), 7.00 (d, *J* = 8.0 Hz, 1H), 7.08–7.12 (m, 3H), 7.33 (s, 1H), 7.41 (d, *J* = 7.6 Hz, 1H), 7.67 (s, 4H), 8.35 (dd, *J*_1_ = 1.6 Hz, *J*_2_ = 4.8 Hz, 1H); HRMS (ESI): calcd for C_29_H_30_ClN_3_O_3_S [M + H]^+^ *m/z*: 536.1769, found: 536.1771.

*N-(4-(4-(8-Chloro-5H-benzo[5,6]cyclohepta[1,2-b]pyridin-11(6H)-ylidene)piperidine-1-carbonyl) phenyl)benzamide* (**1g**). Yield: 85%, m.p.: 128.5–131.6 °C; ^1^H-NMR (CDCl_3_): *δ* 2.41–2.53 (m, 4H), 2.78–2.88 (m, 2H), 3.31–3.42 (m, 4H), 3.68 (s, 1H), 4.14 (s, 1H), 7.12–7.17 (m, 4H), 7.36 (d, *J* = 8.8Hz, 2H), 7.43–7.55 (m, 4H), 7.64 (d, *J* = 8.8 Hz, 2H), 7.88 (dd, *J*_1_ = 1.2 Hz, *J*_2_ = 7.2 Hz, 2H), 8.34 (s, 1H), 8.40 (s, 1H); HRMS (ESI): calcd for C_33_H_28_ClN_3_O_2_ [M + H]^+^ *m/z*: 534.1943, found: 534.1939.

*N-(4-(4-(8-Chloro-5H-benzo[5,6]cyclohepta[1,2-b]pyridin-11(6H)-ylidene)piperidine-1-carbonyl)* *phenyl)-3-methylbenzamide* (**1j**). Yield: 79%; m.p.: 154.9–156.0 °C; ^1^H-NMR (CDCl_3_): *δ* 2.40–2.56 (m, 7H), 2.76–2.89 (m, 2H), 3.28–3.43 (m, 4H), 3.68–3.71 (m, 1H), 4.14 (s, 1H), 7.09–7.17 (m, 4H), 7.31–7.45 (m, 5H), 7.63–7.70 (m, 4H), 8.28 (s, 1H), 8.34 (s, 1H); HRMS (ESI): calcd for C_34_H_30_ClN_3_O_2_ [M + H]^+^ *m/z*: 548.2099, found: 548.2108.

*2-Chloro-N-(4-(4-(8-chloro-5H-benzo[5,6]cyclohepta[1,2-b]pyridin-11(6H)-ylidene)piperidine-1-carbonyl)phenyl)benzamide* (**1k**). Yield: 96%, mp: 120.4–123.1 °C; ^1^H-NMR (CDCl_3_): *δ* 2.42–2.58 (m, 4H), 2.79–2.89 (m, 2H), 3.29–3.43 (m, 4H), 3.70 (br s, 1H), 4.14 (br s, 1H), 7.10–7.17 (m, 4H), 7.35–7.46 (m, 7H), 7.65–7.75 (m, 3H), 8.14 (s, 1H), 8.41 (s, 1H); HRMS (ESI): calcd for C_33_H_27_Cl_2_N_3_O_2_ [M + H]^+^ *m/z*: 568.1553, found: 568.1547.

*3-Chloro-N-(4-(4-(8-chloro-5H-benzo[5,6]cyclohepta[1,2-b]pyridin-11(6H)-ylidene)piperidine-1-carbonyl)phenyl)benzamide* (**1l**). Yield: 70%, m.p.: 241.8–243.7 °C; ^1^H-NMR (CDCl_3_): *δ* 2.43–2.54 (m, 4H), 2.77–2.90 (m, 2H), 3.31–3.44 (m, 4H), 3.69–3.73 (m, 1H), 4.11–4.14 (m, 1H), 7.01–7.18 (m, 4H), 7.39–7.58 (m, 7H), 7.79 (d, *J* = 8.0 Hz, 1H), 7.92 (t, *J* = 2.0 Hz, 1H), 8.39 (s, 1H); HRMS (ESI): calcd for C_33_H_27_Cl_2_N_3_O_2_ [M + H]^+^ *m/z*: 568.1553, found: 568.1556.

*N-(4-(4-(8-Chloro-5H-benzo[5,6]cyclohepta[1,2-b]pyridin-11(6H)-ylidene)piperidine-1-carbonyl)* *phenyl)-2-fluorobenzamide* (**1m**). Yield: 94%, m.p.: 166.9–167.8 °C; ^1^H-NMR (CDCl_3_): *δ* 2.41–2.56 (m, 4H), 2.77–2.90 (m, 2H), 3.32–3.44 (m, 4H), 3.71 (br s, 1H), 4.16 (br s, 1H), 7.09–7.23 (m, 5H), 7.30–7.32 (m, 1H), 7.44–7.46 (m, 3H), 7.51–7.57 (m, 1H), 7.70 (d, *J* = 8.4 Hz, 2H), 8.14–8.19 (m, 1H), 8.40 (s, 1H), 8.54 (d, *J* = 15.6 Hz, 1H); HRMS (ESI): calcd for C_33_H_27_ClFN_3_O_2_ [M + H]^+^ *m/z*: 552.1849, found: 552.1841.

*N-(4-(4-(8-Chloro-5H-benzo[5,6]cyclohepta[1,2-b]pyridin-11(6H)-ylidene)piperidine-1-carbonyl)* *phenyl)-3-methoxybenzamide* (**1n**). Yield: 52%, m.p.: 148.3–151.0 °C; ^1^H-NMR (CDCl_3_): *δ* 2.39–2.59 (m, 4H), 2.77–2.89 (m, 2H), 3.28–3.43 (m, 4H), 3.71 (br s,1H), 3.84 (s, 3H), 4.17 (br s, 1H), 7.05–7.20 (m, 5H), 7.33–7.45 (m, 6H), 7.63 (d, *J* = 8.8 Hz, 2H), 8.31 (s, 1H), 8.39 (s, 1H); HRMS (ESI): calcd for C_34_H_30_ClN_3_O_3_ [M + H]^+^ *m/z*: 564.2048, found: 564.2049.

*N-(4-(4-(8-Chloro-5H-benzo[5,6]cyclohepta[1,2-b]pyridin-11(6H)-ylidene)piperidine-1-carbonyl) phenyl)-3-nitrobenzamide* (**1o**). Yield: 53%, m.p.: >250 °C; ^1^H-NMR (CDCl_3_): *δ* 2.32–2.45 (m, 4H), 2.75–2.89 (m, 2H), 3.25–3.42 (m, 4H), 3.62 (br s, 1H), 4.16 (br s, 1H), 7.09–7.24 (m, 6H), 7.43 (d, *J* = 8.4 Hz, 3H), 7.61–7.65 (m, 1H), 8.32–8.41 (m, 3H), 8.86 (t, *J* = 2.0 Hz,1H), 9.37 (s, 1H); HRMS (ESI): calcd for C_33_H_27_ClN_4_O_4_ [M + H]^+^ *m/z*: 579.1794, found: 579.1786.

*N-(4-(4-(8-Chloro-5H-benzo[5,6]cyclohepta[1,2-b]pyridin-11(6H)-ylidene)piperidine-1-carbonyl) phenyl)-4-nitrobenzamide* (**1p**)*.* Yield: 68%, m.p.: >250 °C; ^1^H-NMR (CDCl_3_): *δ* 2.27–2.56 (m, 4H), 2.79–2.91 (m, 2H), 3.32–3.43 (m, 4H), 3.66 (br s, 1H), 4.16 (br s, 1H), 7.12–7.18 (m, 4H), 7.37–7.46 (m, 3H), 7.60 (d, *J* = 8.0 Hz, 2H), 8.10 (d, *J* = 8.4 Hz, 2H), 8.32–8.37 (m, 4H); HRMS (ESI): calcd for C_33_H_27_ClN_4_O_4_ [M + H]^+^ *m/z*: 579.1794, found: 579.1792.

*N-(4-(4-(8-Chloro-5H-benzo[5,6]cyclohepta[1,2-b]pyridin-11(6H)-ylidene)piperidine-1-carbonyl)* *phenyl)-4-methylbenzenesulfonamide* (**1q**). Yield: 92%, m.p.: 161.5–162.9 °C; ^1^H-NMR (CDCl_3_): *δ* 2.33–2.50 (m, 7H), 2.78–2.86 (m, 2H), 3.28–3.39 (m, 4H), 3.58 (br s, 1H), 4.13 (br s, 1H), 7.04–7.25 (m, 10 H), 7.42 (d, *J* = 6.4 Hz, 1H), 7.59–7.65 (m, 3H), 8.36 (s, 1H); HRMS (ESI): calcd for C_33_H_30_ClN_3_O_3_S [M + H]^+^ *m/z*: 584.1769, found: 584.1777.

*2-Chloro-N-(4-(4-(8-chloro-5H-benzo[5,6]cyclohepta[1,2-b]pyridin-11(6H)-ylidene)piperidine-1-carbonyl)phenyl)benzenesulfonamide* (**1r**). Yield: 80%, m.p.: 153.9–155.5 °C; ^1^H-NMR (CDCl_3_): *δ* 2.39–2.46 (m, 4H), 2.73–2.87 (m, 2H), 3.17–3.39 (m, 4H), 3.57 (br s, 1H), 4.09 (br s, 1H), 7.06–7.14 (m, 6H), 7.23–7.34 (m, 3H), 7.40–7.45 (m, 3H), 7.630 (s, 1H), 8.01 (d, *J* =8.4 Hz, 1H), 8.36 (s, 1H); HRMS (ESI): calcd for C_32_H_27_Cl_2_N_3_O_3_S [M + H]^+^ *m/z*: 604.1223, found: 604.1224.

*2,5-Dichloro-N-(4-(4-(8-chloro-5H-benzo[5,6]cyclohepta[1,2-b]pyridin-11(6H)-ylidene)piperidine-1-carbonyl)phenyl)benzenesulfonamide* (**1s**). Yield: 96%, m.p.: 147.8–149.5 °C; ^1^H-NMR (CDCl_3_): *δ* 2.15–2.40 (m, 4H), 2.74–2.87 (m, 2H), 3.20–3.52 (m, 5H), 4.11 (br s, 1H), 7.08–7.11 (m, 6H), 7.24–7.28 (m, 2H), 7.34–7.43 (m, 3H), 7.51–7.53 (m, 1H), 7.98 (d, *J* =2.0 Hz, 1H), 8.38 (s, 1H); HRMS (ESI): calcd for C_32_H_26_Cl_3_N_3_O_3_S [M + H]^+^ *m/z*: 640.0833, found: 640.0824.

*N-(3-((4-(8-Chloro-5H-benzo[5,6]cyclohepta[1,2-b]pyridin-11(6H)-ylidene)piperidin-1-yl)sulfonyl) phenyl)benzamide* (**1t**). Yield: 78%, m.p.: 119.8–122.4 °C; ^1^H-NMR (CDCl_3_): *δ* 2.34–2.37 (m, 2H), 2.44–2.50 (m, 1H), 2.56–2.63 (m, 1H), 2.68–2.81 (m, 2H), 2.88–2.94 (m, 2H), 3.20–3.39 (m, 4H), 3.89 (s, 1H), 6.83 (dd, *J*_1_ = 2.0 Hz, *J*_2_ = 8.0 Hz, 1H), 6.99–7.01 (m, 2H), 7.04–7.12 (m, 4H), 7.23–7.27 (m, 1H), 7.38 (dd, *J*_1_ = 1.2 Hz, *J*_2_ = 7.6 Hz, 1H), 8.34 (dd, *J*_1_ = 2.0 Hz, *J*_2_ = 4.8 Hz, 1H); HRMS (ESI): calcd for C_32_H_28_ClN_3_O_3_S [M + H]^+^ *m/z*: 570.1613, found: 570.1612.

*3-Chloro-N-(3-((4-(8-chloro-5H-benzo[5,6]cyclohepta[1,2-b]pyridin-11(6H)-ylidene)piperidin-1-yl)-sulfonyl)phenyl)benzamide* (**1u**). Yield: 70%, m.p.: 131.2–133.5 °C; ^1^H-NMR (CDCl_3_): *δ* 2.31–2.36 (m, 2H), 2.42–2.49 (m, 1H), 2.55–2.62 (m, 1H), 2.67–2.81 (m, 2H), 2.85–2.91 (m, 2H), 3.19–3.38 (m, 4H), 6.98 (d, *J* = 8.0 Hz, 1H), 7.04–7.11 (m, 3H), 7.37–7.54 (m, 5H), 7.77 (d, *J* = 7.6 Hz, 1H), 7.90 (dd, *J*_1_ = 1.6 Hz, *J*_2_ = 10.0 Hz, 1H), 8.10 (dd, *J*_1_ = 2.0 Hz, *J*_2_ = 7.2 Hz, 1H), 8.19 (s, 1H), 8.33 (d, *J* = 3.6 Hz, 1H); HRMS (ESI): calcd for C_32_H_27_Cl_2_N_3_O_3_S [M + H]^+^ *m/z*: 604.1223, found: 604.1218.

*N-(3-((4-(8-Chloro-5H-benzo[5,6]cyclohepta[1,2-b]pyridin-11(6H)-ylidene)piperidin-1-yl)sulfonyl) phenyl)-3-nitrobenzamide* (**1v**). Yield: 87%, m.p.: 206.1–208.2 °C; ^1^H-NMR (CDCl_3_): *δ* 2.34–2.43 (m, 2H), 2.56–2.62 (m, 1H), 2.80–2.84 (m, 2H), 2.94–3.07 (m, 2H), 3.29–3.53 (m, 4H), 4.19 (s, 1H), 7.16–7.23 (m, 3H), 7.54–7.57 (m, 2H), 7.66–7.70 (m, 2H), 8.07 (d, *J* = 7.6 Hz, 1H), 8.20 (s, 1H), 8.35 (dd, *J*_1_ = 2.0 Hz, *J*_2_ = 8.0 Hz, 1H), 8.51–8.65 (m, 3H), 9.17 (s, 1H); HRMS (ESI): calcd for C_32_H_27_ClN_4_O_5_S [M + H]^+^ *m/z*: 615.1464, found: 615.1461.

*N-(3-((4-(8-Chloro-5H-benzo[5,6]cyclohepta[1,2-b]pyridin-11(6H)-ylidene)piperidin-1-yl)sulfonyl)* *phenyl)-4-methylbenzenesulfonamide* (**1w**). Yield: 98.4%; m.p.: 192.7–194.9 °C; ^1^H-NMR (CDCl_3_): *δ* 2.26–2.28 (m, 2H), 2.31 (s, 3H), 2.50–2.54 (m, 1H), 2.77–2.83 (m, 2H), 2.94–3.03 (m, 2H), 3.10 (s, 1H), 3.22–3.25 (m, 1H), 3.32–3.36 (m, 1H), 3.46–3.50 (m, 1H), 3.88 (s, 1H), 7.14–7.22 (m, 4H), 7.33–7.43 (m, 2H), 7.55 (d, *J* = 8.0 Hz, 1H), 7.61 (s, 1H), 7.66 (t, *J* = 6.4 Hz, 1H), 7.75 (d, *J* = 8.0 Hz, 2H), 8.04 (d, *J* = 8.0 Hz, 1H), 8.61 (d, *J* = 4.8 Hz, 1H), 9.14 (s, 1H); HRMS (ESI): calcd for C_32_H_30_ClN_3_O_4_S_2_ [M + H]^+^ *m/z*: 620.1439, found: 620.1439.

*3-Chloro-N-(4-((4-(8-chloro-5H-benzo[5,6]cyclohepta[1,2-b]pyridin-11(6H)-ylidene)piperidin-1-yl)-sulfonyl)phenyl)benzamide* (**1x**). Yield: 96%; m.p.: 130.5–133.0 °C; ^1^H-NMR (CDCl_3_): *δ* 2.32–2.38 (m, 2H), 2.45–2.47 (m, 1H), 2.58–2.59 (m, 1H), 2.71–2.89 (m, 4H), 3.22–3.33 (m, 4H), 6.99 (d, *J* = 8.4 Hz, 1H), 7.06–7.11 (m, 3H), 7.39–7.45 (m, 2H), 7.52–7.55 (m, 1H), 7.70–7.87 (m, 6H), 8.13 (s, 1H), 8.34 (dd, *J*_1_ = 1.6 Hz, *J*_2_ = 4.8 Hz, 1H); HRMS (ESI): calcd for C_32_H_27_Cl_2_N_3_O_3_S [M + H]^+^ *m/z*: 604.1223, found: 604.1219.

*N-(4-((4-(8-Chloro-5H-benzo[5,6]cyclohepta[1,2-b]pyridin-11(6H)-ylidene)piperidin-1-yl)sulfonyl) phenyl)-4-methylbenzenesulfonamide* (**1y**). Yield: 78%; m.p.: 126.1–129.1 °C; ^1^H-NMR (CDCl_3_): *δ* 2.29–2.35 (m, 2H), 2.38 (s, 3H), 2.43–2.46 (m, 1H), 2.57–2.59 (m, 1H), 2.70–2.82 (m, 2H), 2.89–2.93 (m, 2H), 3.14–3.30 (m, 4H), 6.98 (d, *J* = 8.0 Hz, 1H), 7.07–7.12 (m, 3H), 7.16–7.19 (m, 2H), 7.26–7.30 (m, 3H), 7.42 (d, *J* = 6.4 Hz, 1H), 7.57–7.60 (m, 2H), 7.73 (d, *J* = 8.4 Hz, 2H), 8.35 (d, *J* = 4.0 Hz, 1H); HRMS (ESI): calcd for C_32_H_30_ClN_3_O_4_S_2_ [M + H]^+^ *m/z*: 620.1439, found: 620.1437.

### 3.3. Biological Evaluation

The *in vitro* evaluation was done by a slightly modified method we reported previously [31]. An *in vitro* radioligand binding assay was performed to determine the binding affinity of the candidates to human V2 and V1a receptors. The functional activity was then subsequently determined by measuring the activation or inhibition of vasopressin induced cAMP accumulation in V2 receptor expressing cells. We investigated some potent derivatives for *in vivo* diuretic activity in conscious hydrated male Sprague-Dawley rats at 8 weeks of age (body weight: (260 ± 20) g). Urine volume was measured 20 h after oral administration of the test compounds.

## 4. Conclusions

Twenty-one derivatives of desloratadine designed as AVP V2 receptor antagonists were synthesized and characterized by ^1^H-NMR, HRMS and HPLC. Their biological activity was evaluated by *in vitro* radioligand binding assay, cAMP assay and *in vivo* diuretic assay. Compounds **1n**, **1t** and **1v** exhibited both high affinity and promising selectivity for V2 receptors. The selected compounds showed promising diuretic results in rats, especially compound **1t**, which produced a total urine volume equivalent to tolvaptan during the experimental period. Through the present studies, compound **1t**, which has good efficacy both *in vitro* and *in vivo*, could be a novel AVP V2 receptor antagonist candidate. Further preclinical studies are however still required.
